# Brazilian primary school teachers' knowledge about immediate management of dental trauma

**DOI:** 10.1590/2176-9451.19.5.110-115.oar

**Published:** 2014

**Authors:** Matheus Melo Pithon, Rogério Lacerda dos Santos, Pedro Henrique Bomfim Magalhães, Raildo da Silva Coqueiro

**Affiliations:** 1 Professor, Department of Orthodontics, State University of Southwestern Bahia (UESB); 2 Professor, Department of Orthodontics, Federal University of Campina Grande (UFCG); 3 DDS, State University of Southwestern Bahia (UESB); 4 Professor, Department of Epidemiology, State University of Southwestern Bahia (UESB)

**Keywords:** Knowledge, Teaching, Dental care

## Abstract

**OBJECTIVE:**

To assess the level of knowledge of primary school teachers in the public school network of Northeastern Brazil with respect to management of dental trauma and its relationship with prognosis.

**METHODS:**

A questionnaire was applied to 195 school teachers of public schools in Northeastern Brazil. The questionnaire comprised 12 objective questions about dental trauma and methods for its prevention and management. Data were submitted to chi-square test and Poisson regression test (P > 0.05).

**RESULTS:**

Out of the 141 teachers who responded the questionnaires, the majority were women (70.2%) and most of them had experienced previous dental accidents involving a child (53.2%). The majority (84.4%) had incomplete college education and few were given some training on how to deal with emergency situations during their undergraduate course (13.5%) or after it (38.3%). Their level of knowledge about dental trauma and emergency protocols showed that unsatisfactory knowledge level was associated with the male sex: 46% higher for men in comparison to women (P = 0.025).

**CONCLUSIONS:**

Approximately half of teachers evaluated had unsatisfactory knowledge about dental trauma and emergency protocols, with female teachers showing more knowledge than men.

## Abstract (pt)

**OBJETIVO:**

avaliar o nível de conhecimento de professores de escolas primárias na rede pública de ensino na região nordeste do Brasil, no que diz respeito ao manejo do paciente traumatizado e sua relação com o prognóstico.

**MÉTODOS:**

aplicou-se um questionário a 195 professores da rede pública de ensino da região nordeste do Brasil. O questionário continha 12 questões objetivas sobre os traumatismos dentários e seus métodos de prevenção e manejo. Após obtenção dos dados, esses foram submetidos ao teste qui-quadradro e ao teste de regressão de Poisson (p > 0,05).

**RESULTADOS:**

dos 141 professores que responderam o questionário, a maioria era composta pelo sexo feminino (70,2%) e a maior parte já vivenciara algum episódio de acidente com criança (53,2%). A maioria (84,4%) possui ensino superior incompleto e poucos foram contemplados com manobras de urgência na graduação (13,5%) ou após essa (38,3%). Os resultados da regressão para o nível de conhecimento sobre traumatismos dentários e protocolo de urgência em relação às variáveis explanatórias do estudo demonstraram que o conhecimento insatisfatório foi associado ao sexo masculino, sendo 46% maior para os homens (p = 0,025).

**CONCLUSÃO:**

aproximadamente metade dos professores possui conhecimento insatisfatório sobre traumatismos dentários e protocolo de urgência, sendo que os professores do sexo feminino apresentaram maior conhecimento do que os do sexo masculino.

## INTRODUCTION

Dentoalveolar trauma is frequent among children and adolescents.^1^^,^ 2 ^,^ 3 It may affect teeth, soft tissues and supporting structures, and may lead to psychological, social, masticatory, phonological and esthetic changes.^4 ^At present, this is considered a public health problem due to the growing rates of violence, automobile accidents, contact sports and injuries in the school environment.^3^^,^ 5 Some studies assert that the number of cases with dental trauma will exceed cases with dental caries or periodontal problems,^6^^,^ 7 and may result in high costs to Public Health Services.^8^

Accidents are the main cause of dental trauma^1^^,^ 9 ^,^ 10 and frequently occur when the child reaches school age. Dental lesions may range from slight to extensive maxillofacial damage.^1^

Parents and teachers who deal with children must be familiarized with dental emergency maneuvers.^1^^,^ 2 However, studies^1^^,^ 3 ^,^ 4 ^,^ 9 ^-^ 12 have shown lack of teacher's knowledge regarding emergency management of dental trauma.^1^ Lack of knowledge on these questions lead to implementation, frequently inadequate, of health policies that do not achieve ideal results.^1^^,^ 2 ^,^ 9 ^,^ 10

Bearing in mind the importance of this issue and the lack of information in Northeastern Brazil, the aim of this study was to investigate the knowledge of school teachers working in the public school network of the municipality of Jéquie / BA about dental injuries caused by trauma, and the procedures to be carried out when they occur.

## MATERIAL AND METHODS

A field research was conducted. Data was collected by means of a questionnaire answered by 195 full-time teachers working in the public school network of the city of Jequié / BA in 2012. Data on the total number of teachers was provided by the Municipal Secretary of Education in the city of Jequié and by the Regional Board of Education (DIREC-13). The questionnaire comprised 12 objective questions and was self-applied in the presence of the main researcher. The first part of the questionnaire consisted in collecting general information about teachers' personal and professional profiles, including age, sex, career time-span, and whether or not they had received any training about dental trauma. The second part consisted of questions with reference to knowledge about dental trauma and dental emergency protocols, hypothesizing situations that could occur in the school environment. To assess teachers' level of knowledge, those who correctly answered 4 to 6 questions were classified as having satisfactory level of knowledge, and those who correctly answered 0 to 3 questions, as having an unsatisfactory level of knowledge. The research project was approved by the Institutional Review Board of UESB, Protocol N^o^.089/011.

The frequency of responses given by the teachers was compared by means of chi-square test (P > 0.05). Associations between the dependent variable (level of knowledge) and explanatory variables (sex, age group, educational level, career time-span, first-aid, dental trauma and first aid training during academic education and having witnessed an accident) were tested by means of Poisson regression technique. Simple robust models were calculated to estimate the prevalence ratios (PR) with their respective confidence interval of 95% (CI: 95%). Significance level was set at 5% (α = 0.05). Data were tabulated and analyzed in Statistical Package for Social Sciences for Windows (SPSS. 15.0, 2006, SPSS, Inc, Chicago, IL, USA) software.

## RESULTS

Teachers' response rate was 72.3% (n = 141). A total of 54 teachers (n = 27.7) decided not to participate in the research. Career time-span ranged from 1 to 33 years, with a mean of 13.5 ± 9.5 years. The majority of teachers (64.5%) aged between 31 and 50 years and had a level of incomplete professional college education (84.4%). They had not had first aid training during their academic education (86.5%) or after it (61.7%), but the majority had witnessed accidents (53.2%) (Table 1).

**Table 1:** Characteristics of study participants.

Characteristics	n	%
**Sex**		
Male	42	29.8
Female	99	70.2
**Age group**		
≤ 30 years	34	24.1
31 to 40 years	46	32.6
41 to 50 years	45	31.9
> 50 years	16	11.3
**Educational level**		
Incomplete college education	119	84.4
Complete college education	22	15.6
Career time-span*		
≤ 6 years	49	34.8
7 to 19 years	47	33.3
> 19 years	45	31.9
**First aid training**		
Yes	54	38.3
No	87	61.7
**Dental trauma and first aid information during academic education**		
Yes	19	13.5
No	122	86.5
**Witnessed accident**		
Yes	75	53.2
No	66	46.8

* For categorization of career time-span, distribution into terciles was taken into consideration: 1st tercile = 6 years and 2nd tercile = 19 years.

**Figure 1:**
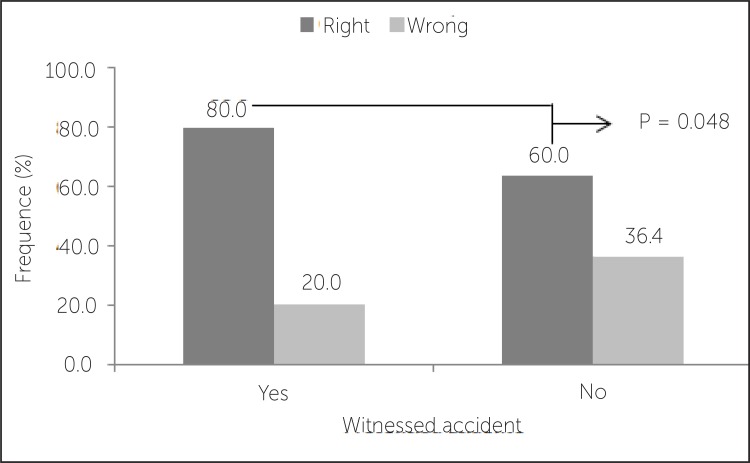
Teachers distribution according to knowledge about the type of tooth fractured and whether or not they witnessed an accident.

Associations between knowledge (Table 2) and the variables presented in Table 1 were tested. Chi-square test highlighted a single association: Knowledge about the type of tooth (Question 1) *vs*. witnessed an accident. Results showed that teachers who had witnessed some type of accident had a higher frequency of correct answers in comparison to those who had never witnessed one (P = 0.048) (Fig 1). For the other questions, no statistical differences were observed. (P > 0.05).

**Table 2:** Teachers distribution with regard to knowledge of dental trauma and emergency protocol.

Question	Correct	Incorrect
**1. A 9-year-old child is hit on the face by a ball and fractures two anterior teeth. Are the affected teeth:**		
( ) Permanent teeth. ( ) Milk teeth.	102 (72.3%)	39 (27.7%)
**2. Which of the following actions do you consider most adequate?**		
( ) You will look for the parts of broken tooth and after class, would contact his parents to explain what had happened. ( ) You will look for the parts of broken tooth and then give him a warm drink and would contact her parents. ( ) You will look for the parts of broken tooth and would contact his parents and then send him immediately to the dentist.	83 (58.9%)	58 (41.1%)
**3. At school, a 12-year-old child falls down the stairs and hits his/her mouth on the floor. One of his/her top front teeth was knocked out of the mouth. What would be the first thing you do?**		
( ) You would look for the tooth and wash it with tap water. ( ) You would ask the child to bite on a tissue paper to control bleeding. ( ) You would ask the child to hold the tooth carefully in his mouth and take her immediately to the nearest dentist. ( ) You would look for the tooth and put it back into the socket.	70 (49.6%)	71 (50.4%)
**4. If you decide to reimplant the tooth back in its place, but it had fallen on the floor, what would you do?**		
( ) You would scrub the tooth gently with a toothbrush. ( ) You would rinse the tooth under tap water. ( ) You would put the tooth straight back into the socket without any pretreatment.	108 (76.6%)	33 (23.4%)
**5. If you chose to wash the tooth, which solution would you use to wash it?**		
( ) Tap water. ( ) Saline solution. ( ) Alcohol. ( ) Filtered water. ( ) Antiseptic solution.	103 (73.0%)	38 (27.0%)
**6. If you do not reimplant the tooth, how would you transport it to the dentist?**		
( ) Tap water. ( ) Milk. ( ) Child’s mouth. ( ) Paper tissue. ( ) Filtered water.	24 (17.0%)	117 (83.0%)

**Figure 2:**
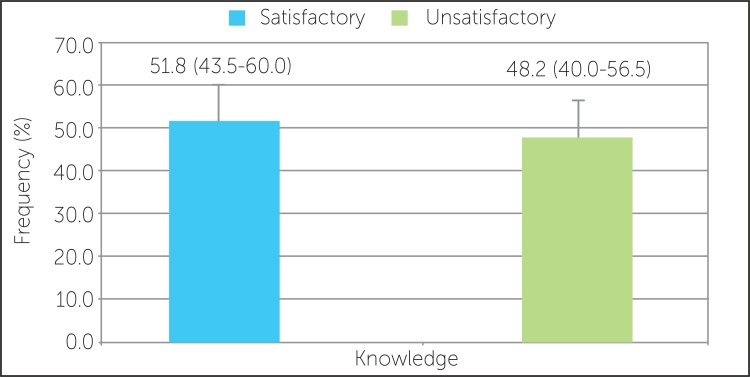
Teachers distribution [prevalence (CI 95%)] according to level of knowledge about dental trauma and emergency protocols.

In the six questions asked, the mean score for right answers was 3.5 ± 1.2 questions. Results revealed that nearly half of teachers had unsatisfactory knowledge with respect to dental trauma and emergency protocols (Fig 2).

The results of regression for the level of knowledge about dental trauma and emergency protocols as regards the explanatory variables of the study (Table 3), demonstrated that unsatisfactory level of knowledge was associated with the male sex: 46% higher for men in comparison to women (P = 0.025). The other variables were not associated with teachers' level of knowledge (P > 0.05).

**Table 3:** Association between the level of unsatisfactory knowledge about dental trauma/emergency protocols and characteristics of the studied sample.

Variables	%	PR (CI 95%)	P value
**Sex**			
Male	61.9	1.46 (1.05 – 2.03)	0.025
Female	42.4	1	
**Age group**			
≤ 30 years	50.0	1.00 (0.55 – 1.81)	0.721
31 to 40 years	41.3	0.83 (0.45 – 1.50)	
41 to 50 years	53.3	1.07 (0.61 – 1.87)	
> 50 years	50.0	1	
**Educational level**			
Incomplete college education	54.5	1.16 (0.56 – 1.32)	0.497
Complete college education	47.1	1	
**Career time-span**			
≤ 6 years	49.0	1.05 (0.69 – 1.60)	0.969
7 a 19 years	48.9	1.05 (0.68 – 1.61)	
> 19 years	46.7	1	
**First aid training**			
Yes	46.3	1	0.720
No	49.4	1.07 (0.75 – 1.53)	
**Learning about dental trauma and first aid in academic education**			
Yes	47.4	1	0.936
No	48.4	1.02 (0.61 – 1.70)	
**Witnessed an accident**			
Yes	46.7	1	0.692
No	50.0	1.07 (0.76 – 1.51)	

PR, prevalence ratio; CI 95%, confidence interval at 95%.

## DISCUSSION

At least half of schoolchildren face the possibility of suffering dentoalveolar trauma during school time.^3^^,^ 13 Dental trauma is relevant in children and adolescents, since their permanent teeth are erupting at this phase.^14^ Additionally, at school, during sporting and recreational activities, children and adolescents are the main groups with an increased likelihood of dental trauma,^3^^,^ 5 ^,^ 15 thereby rendering investigation of school teachers knowledge with regard to dental injuries and treatment approaches.^1^^,^ 2 ^,^ 11 ^,^ 12 ^,^ 15 ^-^ 21

In the present study, approximately half teachers surveyed (48.2%) had unsatisfactory knowledge (correctly answered up to three questions) about dental trauma and emergency protocols. Their mean career time-span was 13.5 years. Only 38.3% of teachers had received first aid training on dental trauma, which was higher than the percentage found by Al-Obaida^1^ who showed only 1.5%. A total of 53.2% teachers who had received training experienced some type of accident involving a child in the school environment, a higher percentage than the 20% found by Arikan.^2^

As for the most adequate solution for washing an avulsed tooth,^22^ 73% of the teachers answered the question correctly by stating filtered water or saline solution; however, only 17% correctly stated milk, the oral cavity, or filtered water as being adequate for sending the avulsed tooth to the dentist.^22^ These findings are similar to those observed in studies conducted in other countries^1^^,^ 2 ^,^ 11 ^,^ 12 ^,^ 16 ^-^ 21 and are important for defining educational strategies, because storing a tooth in an inappropriate environment, in addition to rapidly transporting the child and the tooth to a dentist is crucial for favorable prognosis.^2^^,^ 15 ^,^ 23

Variables such as age, educational level, career time-span, having undergone a first aid training course, having received training on how to deal with emergency situations during their undergraduate course, and having witnessed an accident did not result in greater knowledge about dental trauma and emergency protocols.^2^^,^ 11 ^,^ 15 Female teachers had more knowledge about dental trauma and emergency protocols in comparison to male teachers. This may be related to the fact that women have more contact with children in outdoor environments, in addition to the fact that the majority of them were mothers.

Freitas et al^24^ showed evidence of great lack of knowledge about dentoalveolar trauma in Physical Education professionals, and indicated that they should be better informed on the subject, as they will have to deal with risk situations related to dentoalveolar trauma on a daily basis.^7^^,^ 8 ^,^ 13 ^,^ 22

Children spend great part of their time at school where sporting activities become predisposing factors for dental trauma.^15^ Thus, including emergency procedures in the curriculum of these professionals and implementing educational preventive programs is necessary,^2^^,^ 15 as favorable prognosis will depend on how these injuries are managed.^9^ Therefore, multidisciplinary interaction between dentists and teachers in the public school network is necessary for positive interference in health promotion and prevention of more severe complications.^9^^,^ 25 This includes the dissemination of posters, leaflets, and information through lectures,^2^^,^ 19 television, magazines, radio and newspapers,^10^ or the Internet (http://www.iadt-dentaltrauma.org.).^2^

An educational program^1^^,^ 15 that discusses the importance of preventing dental trauma and the benefits of immediate treatment, conservation of fractures or avulsed teeth would significantly reduce dentoalveolar trauma and sequelae.^26^

Another relevant factor is knowledge about primary and permanent dentitions, and their period of transition.^27 ^In the present study, nearly 28% of teachers were unable to differentiate a permanent to a primary anterior tooth in a 9 year-old-child. A large portion of the population is not aware of the period of primary dentition in a child's development. Early loss of a primary tooth due to trauma may affect the physiological sequence of permanent teeth, and may be etiological factors for malocclusions,^14^ thus stimulating incorrect exercise of perioral musculature and/or cause phonological changes related to teeth.^5^^,^ 11 ^,^ 14

Studies^20^^,^ 28 have shown that teachers with a rudimentary level of learning about dental trauma expressed the desire to receive more information about the subject, totaling 95% of respondents. On the other hand, many primary schools in Japan have nurse teachers with knowledge of emergency care, which is considered a good approach when dealing with children and adolescents.^29^

Knowledge about teachers' ability in dealing with traumatized patients in Northeastern Brazil will make it possible to conduct adequate programs for guidance, prevention^3^ and management of dental trauma, thereby improving prognosis in cases of dental trauma.^2^^,^ 4 ^,^ 9 ^,^ 10 ^,^ 14

## CONCLUSION

Based on the results of this study it is reasonable to conclude that:

» Approximately half of teachers has unsatisfactory knowledge about dental trauma and emergency protocols.
» Female teachers had more knowledge about dental trauma and emergency protocols than male teachers.
» Being older, having a better educational level, longer career time-span, having undergone first aid training related to dental trauma during academic education, and having witnessed an accident did not provide more knowledge of dental trauma and emergency protocols.
